# Beyond Behavior Reduction: A Clinical Perspective on Integrating Developmental, Psychological, and Family-Centered Frameworks into Community ABA Implementation

**DOI:** 10.3390/bs16071076

**Published:** 2026-07-01

**Authors:** Andres Feliciano Mambuca

**Affiliations:** Mental Health Institute of Florida, Pembroke Pines, FL 33028, USA; clinicaldirector@mhiof.com

**Keywords:** applied behavior analysis, autism spectrum disorder, neurodivergence, family systems, emotional regulation, adaptive functioning, caregiver preparation, interdisciplinary care, long-term outcomes

## Abstract

Applied Behavior Analysis (ABA) is recognized as among the most empirically supported interventions for autism spectrum disorder (ASD) and related neurodevelopmental conditions. This clinical perspective examines the gap between controlled research efficacy and community-based implementation by integrating insights from developmental psychology, family systems theory, and implementation science. The paper argues that several limitations sometimes observed in long-term functional outcomes are better understood as challenges of community-level implementation rather than deficiencies of behavioral principles themselves. Furthermore, dimensions occasionally described as “missing” from behavioral practice—including attention to covert behavior, family systems, emotional regulation, and lifespan adaptation—already fall within contemporary best practice, though they may be applied inconsistently across diverse service contexts. Rather than positioning these dimensions as external to behavior analysis, this paper proposes an explicitly interdisciplinary, family-centered, and lifespan-oriented framework in which behavioral science is embedded within structured caregiver preparation, clear referral pathways, and neurodiversity-affirming goals. Key recommendations include flexible service delivery models aligned with family realities; integrated family stress screening and mental-health referral pathways; workforce training in developmental psychology and emotional regulation; and lifespan-oriented outcome measurement beyond behavior reduction. The paper concludes that ABA’s contributions to autism intervention are profound and not in dispute; rather, the field benefits from expanding implementation contexts to address the full scope of neurodevelopmental care and family support. This perspective is intended to generate empirical investigation of proposed service-design innovations and to inform clinical practice in community-based ABA settings serving diverse, underserved populations.

## 1. Introduction

Applied Behavior Analysis has long been recognized as one of the most empirically supported interventions for individuals with autism spectrum disorder (ASD) and other neurodevelopmental conditions. Grounded in learning theory and operant principles, ABA has clarified how environmental contingencies shape behavior and how systematic intervention can reduce behaviors of concern while building adaptive repertoires (Baer et al., 1968; Cooper et al., 2020; Skinner, 1953). Across decades of research, behavior-analytic interventions have improved communication, reduced aggression and self-injury, and promoted learning across developmental domains, particularly when delivered with fidelity during early development (Reichow et al., 2014; Schreibman et al., 2015). These contributions are substantial and are not in dispute here.

At the same time, both clinical practice and the developmental literature have raised questions about the variability of long-term functional outcomes among individuals who receive autism services (Howlin & Moss, 2012; Lord et al., 2020). Reductions in behaviors of concern and gains in targeted skills remain clinically meaningful, yet attention has broadened toward dimensions such as emotional well-being, quality of life, adaptive independence, social participation, and adult outcomes (Mundy, 2018; Pellicano & den Houting, 2022). Importantly, this broadening of focus is not unique to autism services; co-occurring mental health conditions are common across neurodevelopmental and general populations and frequently emerge in adolescence and young adulthood regardless of the early intervention received (Lai et al., 2019).

This paper makes two central arguments. First, many of the difficulties sometimes attributed to behavioral care may arise from the complexity of translating controlled research into community-based implementation. Second, several dimensions sometimes described as absent from behavioral practice—including attention to covert behavior, family systems, emotional regulation, and long-term developmental outcomes—already fall within contemporary best practice, though they may be applied unevenly across diverse service contexts.

### Aims and Scope

This paper is a clinical perspective and does not constitute a systematic or comprehensive review of the literature. It does not follow PRISMA reporting standards, and the clinical impressions it describes are not offered as systematic evidence. Its aims are (1) to articulate where community implementation of ABA may diverge from both controlled-research conditions and best-practice standards; (2) to argue that family and psychological dimensions are central, not peripheral, to developmental outcomes; and (3) to outline a concrete, interdisciplinary, family-centered, and lifespan-oriented model for discussion. Each substantive claim is framed as a hypothesis warranting empirical investigation.

For clarity, several constructs are used throughout with the following working meanings. *Behavior reduction* refers to decreasing behaviors of concern such as aggression or self-injury. *Adaptive functioning* refers to the practical skills needed for everyday independence. *Adaptive adulthood* refers to functioning across employment, independent living, relationships, and self-determination. *Quality of life* refers to subjective well-being and objective life conditions, including emotional health and social participation. *Meaningful participation* refers to inclusion in valued family and community roles consistent with the individual’s preferences and capacities.

## 2. From Controlled Efficacy to Community Implementation

The scientific foundations of ABA rest on well-established principles of reinforcement, stimulus control, prompting, shaping, and environmental modification (Baer et al., 1968; Cooper et al., 2020). Under conditions of strong treatment fidelity, active supervision, and individualized programming, behavior-analytic interventions can produce meaningful gains in communication, adaptive functioning, and skill acquisition (Reichow et al., 2014; Schreibman et al., 2015). Notably, contemporary best-practice models already address several concerns commonly raised about behavioral care: naturalistic developmental behavioral interventions integrate developmental science with behavioral methods, and high-quality programming explicitly targets generalization, the fading of prompts, and reinforcement that approximates natural contingencies (Schreibman et al., 2015).

Translation into large-scale community practice, however, occurs in environments that differ substantially from controlled research settings. Implementation science consistently shows that outcomes depend not only on the intervention model but on contextual variables—family readiness, environmental consistency, provider training, supervision quality, and systemic support (Fixsen et al., 2005). Ecological and transactional models likewise hold that development cannot be separated from the relational and environmental systems in which it unfolds (Bronfenbrenner, 1979; Sameroff, 2009). It is therefore plausible—though it requires empirical confirmation—that variability in long-term outcomes reflects variability in implementation conditions more than limitations of behavioral theory.

Within some home-based service models, intervention unfolds in households contending with significant practical, emotional, and financial demands. Under such pressures, families may understandably prioritize immediate stabilization, and the functional role of therapy in the home may, in some cases, drift toward supervision or support rather than a comprehensive developmental process. These dynamics are not failures of caregiving; they reflect the realities of overwhelmed systems. Where they occur, however, they may reduce opportunities for generalization and sustained developmental growth. Maintaining the original clinical intent is, in best practice, the shared responsibility of the supervising behavior analyst and the caregiver, supported by consistent contact and clear explanation of the rationale behind each procedure—precisely the conditions associated with stronger generalization (Bearss et al., 2015; Oono et al., 2013).

## 3. The Family System as a Central Variable

Although most treatment models recognize the importance of caregiver participation, family involvement is sometimes conceptualized as support for intervention rather than as a central determinant of outcomes. Developmental, ecological, and family-systems frameworks suggest the opposite: child functioning is embedded in the relational and emotional environment of the family (Bronfenbrenner, 1979; Minuchin, 1974). Parent-mediated intervention research consistently associates active caregiver involvement with improved generalization, communication, and maintenance of gains (Bearss et al., 2015; Oono et al., 2013).

Families of neurodivergent children frequently experience elevated stress, emotional exhaustion, and uncertainty about the future relative to families of neurotypical children (Hayes & Watson, 2013; Karst & Van Hecke, 2012). Many also enter services while still processing the meaning of the diagnosis itself. These realities matter because the emotional condition of the caregiving system shapes the consistency of the developmental environment.

A reasonable objection, raised from within behavior analysis, is that intervention must begin somewhere, and that teaching caregivers concrete strategies—reinforcement, token systems, structured routines—has genuine value: a tool that helps a child complete a previously stressful task, or that reduces self-injurious behavior, is a meaningful first step. A caregiver need not fully understand the principles behind a sleep or safety intervention before benefiting from it. This perspective is well taken. The argument here is not that procedural training is unimportant, but that it is a starting point rather than an endpoint. Once behaviors of concern are better managed or replaced with safe, socially valid alternatives, caregivers can be supported toward a deeper understanding of their child’s development—and a skilled analyst characteristically aims to build natural reinforcement, promote generalization across people and settings, and ultimately “work themselves out of a job.” The hypothesis advanced here is that long-term outcomes may improve when this progression toward broader caregiver understanding is treated as an explicit, planned objective rather than an incidental one.

## 4. The Psychological Dimension and the Case for Interdisciplinary Referral

Contemporary developmental and clinical research recognizes that neurodevelopmental conditions involve psychological, emotional, and neurobiological dimensions that extend beyond overt behavior (Lord et al., 2020; White et al., 2009). Elevated rates of anxiety, depression, emotional dysregulation, and related difficulties are well documented among autistic individuals across the lifespan (Kerns et al., 2015; Lai et al., 2019). Behaviors that appear similar on the surface—avoidance, withdrawal, irritability, or noncompliance—may reflect anxiety, sensory overload, executive demands, or emotional exhaustion rather than simple behavioral resistance (Mazefsky et al., 2013; Samson et al., 2014).

It would be inaccurate to suggest that behavior analysis ignores these dimensions. Functional behavioral assessment routinely incorporates setting events—such as poor sleep, illness, or medication changes—and competent practitioners are expected to recognize when a presentation warrants medical or psychological evaluation and to refer accordingly within their scope of competence. Indeed, when manipulation of environmental variables does not produce predictable change, referral for medical or psychiatric assessment is appropriate. The concern, framed here as a clinical hypothesis, is not that these practices are absent from the model but that referral pathways and interdisciplinary collaboration may be inconsistent in community settings, and that direct-service training emphasis varies. This is best understood as a workforce and systems issue rather than a limitation of behavioral science, and it points toward a specific, actionable solution: clearer standards and training for when and how to connect families with appropriate specialists and support networks.

This reframing also clarifies a conceptual point. Integration of psychological and developmental care need not, and should not, occur “through” the behavior analyst alone, whose scope of competence is bounded. A more parsimonious and ethical model places the behavior analyst within an interdisciplinary network—psychology, psychiatry, occupational therapy, speech-language pathology, and education—linked by structured referral and case consultation. The goal is coordinated care, not the expansion of any single discipline beyond its competence.

## 5. Adaptive Outcomes Across the Lifespan

Developmental demands evolve over time. Early childhood intervention often occurs within highly structured environments, and many children make measurable gains in communication, adaptive behavior, and reduction in behaviors of concern (Reichow et al., 2014). Adolescence and adulthood introduce expectations—emotional regulation, social reciprocity, vocational competence, and independent problem-solving—that extend beyond what early structured programming directly targets (Mundy, 2018).

Research on adult outcomes shows substantial variability, with many autistic adults continuing to encounter challenges in employment, independent living, relationships, and well-being (Howlin et al., 2004; Levy & Perry, 2011; Taylor & Seltzer, 2011). Elevated rates of loneliness, social anxiety, and, in some studies, suicidality have been reported, particularly among individuals with greater awareness of social difference (Cassidy et al., 2014; Hedley & Uljarević, 2018). Two cautions are warranted in interpreting these findings. First, such difficulties are not unique to autism or to individuals who received ABA; many co-occurring conditions emerge in young adulthood across populations (Lai et al., 2019). Second, behavioral gains in childhood and adaptive outcomes in adulthood are not equivalent constructs, and the absence of a one-to-one relationship between them should not be read as evidence against behavioral intervention.

What these findings do suggest is that outcome measurement itself may benefit from broadening. Beyond behavior counts and skill acquisition, lifespan-relevant domains include independent-living skills, employment stability, social participation, mental health and psychiatric symptoms, self-determination, interpersonal relationships, caregiver quality of life, and community integration. Where intervention can be designed with these domains in view—building in prompt fading, maximizing independence, and embedding natural reinforcement so that gains persist beyond structured settings—long-term adaptation may be better served. Notably, behavior-analytic principles are not age-bound; they are applied across the lifespan and across populations, including in organizational and adult contexts, which supports their relevance to lifespan planning rather than arguing against it.

## 6. From Procedural Parent Training to Comprehensive Caregiver Preparation

Parent involvement is a well-established contributor to generalization and maintenance of gains (Bearss et al., 2015; Oono et al., 2013). Within many models, however, parent training is conceptualized primarily as instruction in behavioral procedures—reinforcement, prompting, extinction, and token systems. These tools are valuable, but the proposal here is that caregiver preparation be conceptualized more broadly, much as caregiver involvement is understood in occupational, speech, and physical therapy: a small number of weekly clinical hours cannot, on their own, produce durable change, and the caregiver functions as the primary agent of generalization across the day.

Comprehensive caregiver preparation would retain procedural instruction while adding neurodevelopmental psychoeducation, support for emotional adaptation to the diagnosis, family stress regulation, sensory understanding, and preparation for later developmental stages. Table 1 contrasts the two emphases. The distinction is not oppositional; comprehensive preparation includes procedural training and extends it.

## 7. Toward an Integrative, Interdisciplinary, Family-Centered Model

The preceding sections point toward a model that expands the context of behavioral intervention rather than replacing it. Behavioral principles—reinforcement, shaping, prompting, task analysis, functional assessment, and generalization programming—remain foundational (Cooper et al., 2020; Reichow et al., 2014). The proposal is to embed them within a structured, interdisciplinary, and family-centered framework. In concrete terms, such a model would include the following components:

Structured caregiver psychoeducation that pairs procedural training with neurodevelopmental understanding and adjustment support, delivered as a planned curriculum rather than incidental coaching.

Routine family stress and well-being screening at intake and at intervals, with defined thresholds for additional support.

Clear mental-health referral pathways and interdisciplinary case consultation, so that anxiety, depression, trauma, or medical concerns are addressed by appropriate professionals rather than managed solely within behavioral sessions (Kerns et al., 2015; Lai et al., 2019).

Supervision and workforce standards that ensure direct-service providers receive adequate orientation to developmental psychology, emotional regulation, and family context, and that supervisors verify socially valid goals, replacement behaviors for behaviors of concern, and explicit plans for fading support.

Adolescent transition planning that begins well before adulthood and targets autonomy, vocational readiness, and emotional self-regulation.

Lifespan-oriented outcome measurement that supplements behavioral indices with quality-of-life, mental-health, self-determination, and community-integration measures.

A neurodiversity-affirming orientation is integral to this model (see Pellicano & den Houting, 2022). Adaptive skill development should be distinguished from behavioral normalization: the goal is to support authentic development of strengths and adaptive independence, consistent with the individual’s neurodevelopmental profile and preferences.

## 8. Limitations

Several limitations should be stated plainly. This is a single-author clinical perspective, not a systematic review; it does not employ a documented search strategy, inclusion criteria, or formal evidence synthesis, and it is therefore subject to selection bias in the literature cited. The clinical impressions described reflect the author’s experience and are offered as hypotheses, not as generalizable findings; they have not been quantified, and they may not represent practice across the diverse landscape of providers, supervision models, and interdisciplinary programs. Claims regarding variability in provider preparation are advanced as clinical and systems-level concerns requiring empirical workforce research rather than as established conclusions. The model proposed in Section 7 is conceptual and has not been evaluated for feasibility, cost, or effectiveness. 

## 9. Future Directions

The questions raised here translate into concrete research and practice priorities. Longitudinal studies are needed that follow individuals from early intervention into adulthood and that measure quality-of-life, mental-health, employment, and self-determination outcomes alongside behavioral indices. Integrative caregiver-preparation protocols should be developed and tested against standard procedural parent training. Interdisciplinary service models—linking behavior analysis with psychology, psychiatry, and allied health through defined referral pathways—warrant evaluation for feasibility and outcomes. Workforce research should examine the relationship between provider preparation, supervision quality, and long-term results. Finally, the field would benefit from continued work on broadened, consensus definitions of treatment success that incorporate well-being and participation alongside behavior change.

## 10. Conclusions

Applied Behavior Analysis has contributed profoundly to autism intervention, and the present perspective does not dispute that contribution. Its argument is that the complexity of human neurodevelopment calls for embedding behavioral science within a broader developmental, psychological, family-centered, and interdisciplinary framework—and that much of what such a framework requires already exists within best-practice behavior analysis but is delivered inconsistently across community settings. Reductions in behaviors of concern remain clinically important; they are, however, one component of long-term functioning rather than its entirety. The constructive path forward is not to reject behavioral methodology but to implement, consistently and collaboratively, the family-centered, psychologically informed, and lifespan-oriented practices the field already values—so that intervention supports not only behavior change but emotional well-being, adaptive independence, dignity, and meaningful participation across the lifespan.

## Figures and Tables

**Table 1:** Traditional procedural parent training compared with comprehensive caregiver preparation.

Dimension	Traditional Procedural Parent Training	Comprehensive Caregiver Preparation
Primary focus	Correct implementation of behavioral procedures	Sustaining a developmental, emotional, and relational environment over time
Typical content	Reinforcement, prompting, extinction, token systems, data collection	The above, plus neurodevelopmental psychoeducation, emotional adaptation, family regulation, sensory understanding
View of the caregiver	Assistant who implements the protocol	Central developmental partner and primary agent of generalization
Emotional support	Generally minimal or incidental	Explicit: adjustment to diagnosis, caregiver stress, resilience, referral when indicated
Time horizon	Immediate behavior change and skill acquisition	Lifespan: adolescent transition and adult adaptive functioning
Outcome emphasis	Procedural fidelity, behavior reduction, skill counts	Generalization plus well-being, family functioning, and quality of life
Interdisciplinary linkage	Often limited	Built-in referral pathways and case consultation

## Data Availability

No new data were created or analyzed in this study.

## References

[B1-behavsci-16-01076] Baer D. M., Wolf M. M., Risley T. R. (1968). Some current dimensions of applied behavior analysis. Journal of Applied Behavior Analysis.

[B2-behavsci-16-01076] Bearss K., Burrell T. L., Stewart L., Scahill L. (2015). Parent training in autism spectrum disorder: What’s in a name?. Clinical Child and Family Psychology Review.

[B3-behavsci-16-01076] Bronfenbrenner U. (1979). The ecology of human development: Experiments by nature and design.

[B4-behavsci-16-01076] Cassidy S., Bradley P., Robinson J., Allison C., McHugh M., Baron-Cohen S. (2014). Suicidal ideation and suicide plans or attempts in adults with Asperger’s syndrome attending a specialist diagnostic clinic: A clinical cohort study. The Lancet Psychiatry.

[B5-behavsci-16-01076] Cooper J. O., Heron T. E., Heward W. L. (2020). Applied behavior analysis.

[B6-behavsci-16-01076] Fixsen D. L., Naoom S. F., Blase K. A., Friedman R. M., Wallace F. (2005). Implementation research: A synthesis of the literature.

[B7-behavsci-16-01076] Hayes S. A., Watson S. L. (2013). The impact of parenting stress: A meta-analysis of studies comparing the experience of parenting stress in parents of children with and without autism spectrum disorder. Journal of Autism and Developmental Disorders.

[B8-behavsci-16-01076] Hedley D., Uljarević M. (2018). Systematic review of suicide in autism spectrum disorder: Current trends and implications. Current Developmental Disorders Reports.

[B9-behavsci-16-01076] Howlin P., Goode S., Hutton J., Rutter M. (2004). Adult outcome for children with autism. Journal of Child Psychology and Psychiatry.

[B10-behavsci-16-01076] Howlin P., Moss P. (2012). Adults with autism spectrum disorders. Canadian Journal of Psychiatry.

[B11-behavsci-16-01076] Karst J. S., Van Hecke A. V. (2012). Parent and family impact of autism spectrum disorders: A review and proposed model for intervention evaluation. Clinical Child and Family Psychology Review.

[B12-behavsci-16-01076] Kerns C. M., Kendall P. C., Berry L., Souders M. C., Franklin M. E., Schultz R. T., Miller J., Herrington J. (2015). Traditional and atypical presentations of anxiety in youth with autism spectrum disorder. Journal of Autism and Developmental Disorders.

[B13-behavsci-16-01076] Lai M.-C., Kassee C., Besney R., Bonato S., Hull L., Mandy W., Szatmari P., Ameis S. H., Veenstra-VanderWeele J. (2019). Prevalence of co-occurring mental health diagnoses in the autism population: A systematic review and meta-analysis. The Lancet Psychiatry.

[B14-behavsci-16-01076] Levy A., Perry A. (2011). Outcomes in adolescents and adults with autism: A review of the literature. Research in Autism Spectrum Disorders.

[B15-behavsci-16-01076] Lord C., Brugha T. S., Charman T., Cusack J., Dumas G., Frazier T., Jones E. J. H., Jones R. M., Pickles A., State M. W., Taylor J. L., Veenstra-VanderWeele J. (2020). Autism spectrum disorder. Nature Reviews Disease Primers.

[B16-behavsci-16-01076] Mazefsky C. A., Herrington J., Siegel M., Scarpa A., Maddox B. B., Scahill L., White S. W. (2013). The role of emotion regulation in autism spectrum disorder. Journal of the American Academy of Child & Adolescent Psychiatry.

[B17-behavsci-16-01076] Minuchin S. (1974). Families and family therapy.

[B18-behavsci-16-01076] Mundy P. (2018). Autism and joint attention: Development, neuroscience, and clinical fundamentals.

[B19-behavsci-16-01076] Oono I. P., Honey E. J., McConachie H. (2013). Parent-mediated early intervention for young children with autism spectrum disorders. Cochrane Database of Systematic Reviews.

[B20-behavsci-16-01076] Pellicano E., den Houting J. (2022). Annual Research Review: Shifting from “normal science” to neurodiversity in autism science. Journal of Child Psychology and Psychiatry.

[B21-behavsci-16-01076] Reichow B., Barton E. E., Boyd B. A., Hume K. (2014). Early intensive behavioral intervention (EIBI) for young children with autism spectrum disorders. Cochrane Database of Systematic Reviews.

[B22-behavsci-16-01076] Sameroff A. (2009). The transactional model of development: How children and contexts shape each other.

[B23-behavsci-16-01076] Samson A. C., Hardan A. Y., Podell R. W., Phillips J. M., Gross J. J. (2014). Emotion regulation in children and adolescents with autism spectrum disorder. Autism Research.

[B24-behavsci-16-01076] Schreibman L., Dawson G., Stahmer A. C., Landa R., Rogers S. J., McGee G. G., Kasari C., Ingersoll B., Kaiser A. P., Bruinsma Y., McNerney E., Wetherby A., Halladay A. (2015). Naturalistic developmental behavioral interventions: Empirically validated treatments for autism spectrum disorder. Journal of Autism and Developmental Disorders.

[B25-behavsci-16-01076] Skinner B. F. (1953). Science and human behavior.

[B26-behavsci-16-01076] Taylor J. L., Seltzer M. M. (2011). Employment and post-secondary educational activities for young adults with autism spectrum disorders during the transition to adulthood. Journal of Autism and Developmental Disorders.

[B27-behavsci-16-01076] White S. W., Oswald D., Ollendick T., Scahill L. (2009). Anxiety in children and adolescents with autism spectrum disorders. Clinical Psychology Review.

